# MECHANISMS OF GUT MICROBIOTA IN FRAILTY: FINDINGS FROM MENDELIAN RANDOMIZATION AND MOUSE MODEL STUDIES

**DOI:** 10.1093/geroni/igae098.3670

**Published:** 2024-12-31

**Authors:** Ting Xu, Shulin Song, Dan Zhao, Yaguang Zheng, Xiang Qi, Minghui Ji, Qin Xu, Bei Wu

**Affiliations:** nanjing medical university, harrison, New Jersey, United States; nanjing medical university, nanjing, Jiangsu, China (People’s Republic); Shandong University, Jinan, Shandong, China (People’s Republic); New York University, New York, New York, United States; New York University, New York, New York, United States; nanjing medical university, nanjing, Jiangsu, China (People’s Republic); nanjing medical university, nanjing, Jiangsu, China (People’s Republic); New York University, New York, New York, United States

## Abstract

Frailty is a prevalent syndrome among older adults, and recent evidence suggests a connection between gut microbiota and frailty. However, the underlying mechanisms remain unclear. This study aimed to investigate the relationship between gut microbiota and frailty, with a focus on how serum metabolites and inflammatory markers might mediate the association. We used a combination of Mendelian randomization and animal models in our approach. Mice frailty was induced via chronic inflammation and assessed by the frailty index based on mice model. We used high-throughput 16S rDNA sequencing to identify alterations in gut microbiota. Differences between groups were examined through Wilcoxon rank-sum tests. We then used spearman correlation analysis to examine the association between gut microbiota changes and frailty-related indicators and metabolites. MR methods, including inverse variance weighted, MR-Egger, and weighted median approaches were used to estimate causal effects. Our animal experiments revealed gut microbiota dysbiosis in frail mice, notably a reduction in Ruminococcus. We found a positive correlation between Ruminococcus abundance and frailty scores (⍴ = 0.716, p < 0.001) and inflammatory markers. Through metabolomic profiling, we identified several key metabolites potentially involved in frailty, including S-Methyl-5’-thioadenosine, L-anserine, and indoleacetic acid. Further analysis using MR methods confirmed a causal relationship between Ruminococcus obeum abundance and frailty index (p = 0.004, OR = 1.035, 95% CI: [1.011, 1.059]), underscoring the role of inflammatory proteins and metabolites as incomplete mediation. This study provides insights into how gut microbiota contribute to frailty and highlights potential biomarkers and therapeutic targets to support healthy aging.

